# Once a saint, now a sinner: An appropriate or inappropriate shock?

**DOI:** 10.1002/joa3.13209

**Published:** 2025-01-10

**Authors:** Sudipta Mondal, Swasthi S. Kumar, Jyothi Vijay, Narayanan Namboodiri

**Affiliations:** ^1^ Department of Cardiology, Electrophysiology Division Sree Chitra Tirunal Institute for Medical Sciences and Technology Thiruvananthapuram Kerala India

**Keywords:** anti‐tachycardia pacing, ATP, dual tachycardia, implantable cardioverter defibrillator, ventricular tachycardia

## Abstract

Critical analysis of electrograms of any therapy delivery event is paramount to identify the etiology, specificity, and sensitivity of the programmed algorithms to differentiate supraventricular versus ventricular tachycardia, its effectiveness, and potential interventions to prevent recurrence. Besides the aspects mentioned above, this case delves into the potential limitations of existing algorithms and the adverse effects of anti‐tachycardia pacing.

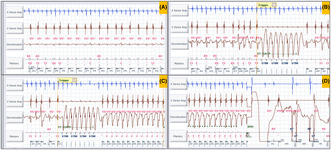

A middle‐aged patient with an implanted dual‐chamber automated implantable cardioverter defibrillator (AICD) for secondary prophylaxis presented to the device clinic with one shock therapy. The patient had a prior history of ischemic heart disease with atrial flutter. The device interrogation tracings are depicted in Figure 1 and File S1. The therapy programming parameters and index event details are depicted in Table 1; and Figure S1. A clinical examination, electrolytes, transthoracic, and transesophageal echocardiogram were within normal limits. What is the likely diagnosis?

**FIGURE 1:**
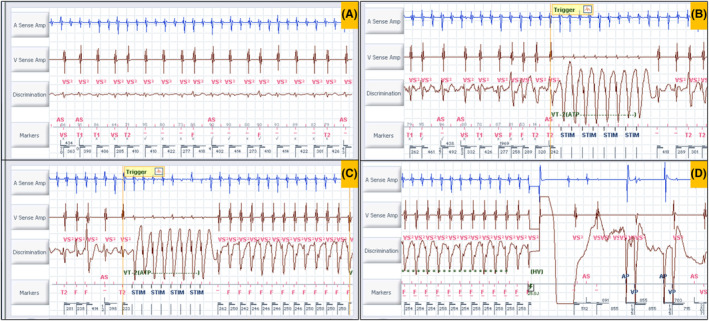
The device interrogation tracings. The gain of the discrimination channel in (A) is one third of the same channel in (B, C, and D).

**TABLE 1:** Therapy zones, parameters, and index therapy details.

Zone	VT‐1	VT‐2	VF
Rate (bpm)	150	181	214
Intervals for binning	18	16	12
Therapy	ATP burst × 3, 25 J, 36 J, 40 J	ATP burst × 3, 25 J, 36 J, 40 J	ATP burst during charging 25 J, 36 J, 40 J × 4
Therapy details	Duration 1:11 [M:S]	Rate branch classification AF/AFL V < A	Stability delta 40 ms
Interval stability ON w/AVA 60 ms	Morphology match 3/10, 90%	Burst 1: 244 ms × 8 Burst 3: 224 ms × 8	One HV shock 25 J

Abbreviations: ATP, anti‐tachycardia pacing; AVA, atrioventricular association; bpm, beats per minute; HV, high voltage; J, Joules; ms, milliseconds; [M:S], [minute: second]; VF, ventricular fibrillation; VT, ventricular tachycardia.

The first intracardiac electrogram (EGM) showed an atrial rate of 250 bpm with variable AV conduction with a tachycardia cycle length (TCL) of 240 ms (Figure 2A). Evaluation of the recording demonstrated a regular atrial rhythm with a rate exceeding that of the ventricular events. Occasional absence of atrial deflections on the marker channel was attributed to the coincidence of atrial depolarization with the post‐ventricular atrial blanking period (PVAB). Far‐field EGM also confirmed the atrial activity (saw‐tooth pattern, File S1) likely being conducted giving rise to a fast ventricular rate. As per the dual‐chamber SVT (supraventricular tachycardia)/VT (ventricular tachycardia) discrimination criteria, it fell in the category of V < A rate branch classification (Figure 3). This classification is followed by the evaluation of (i) interval stability and AV association and (ii) morphology match criteria. AV association criteria comes into effect only when interval stability criteria detects a stable rhythm and considers it as VT. This is to differentiate VT from an atrial tachycardia (AT)/flutter with fixed conduction [2:1 or 3:1]. As per the programming “If any” instead of “if all,” any one of the above would label the tachycardia as VT and therapy would be delivered. Interval stability correctly classified it as SVT because of irregular ventricular rate due to variable conduction. However, the high ventricular rate resulted in aberrant conduction, leading to a mismatch between the far‐field ventricular EGM morphology and the sinus rhythm template, failing to meet at least 3 out of the previous 10 ventricular electrograms the programmed 90% match criteria (Figure 2B). Hence, it was detected as VT and anti‐tachycardia (ATP) pacing was initiated (Figure 2B). However, given the SVT, ATP failed to terminate the same (Figure 2B). Following the third attempt of ATP, the ventricular EGM changed significantly, which was a regular VT at a rate of 240 bpm at TCL of 250 ms (Figure 2C). The EGMs showed AV dissociation confirming the presence of independent atrial and ventricular tachycardia (dual tachycardia). It excludes the possibility of VT with retrograde conduction overdriving the AT. The identified VT fell into the VF zone. Consequently, a shock was delivered, resulting in the termination of both the atrial and ventricular tachycardias (Figure 2D). This case exemplifies the potential hazards of inappropriate ATP therapy for AT with a rapid ventricular rate. In this instance, ATP resulted in the development of dual tachycardia, characterized by the persistence of AT and the induction of VT. Ultimately, both tachycardias were successfully terminated by an appropriately delivered shock.

**FIGURE 2:**
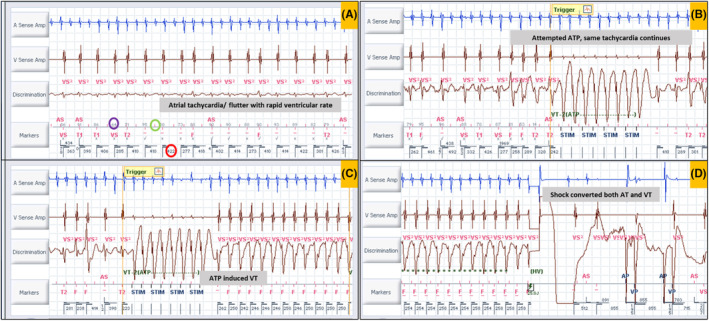
(A) A regular atrial rhythm with a rate exceeding that of the ventricular events (A > V). Red bars (within the green circle) above the marker channel horizontal line indicate atrial events, and the bars below the same line indicate ventricular events. Note that atrial events in the post‐ventricular atrial blanking period (PVAB) are not marked. The values above the line indicate morphology match percentage (purple circle) with the sinus template. If it is <90% match, it is given an “x” mark otherwise, a “✓” mark. Most lower values indicate ventricular cycle length (red circle). The gain of the discrimination channel in (A) is one third of the same channel in (B, C, and D). (B) Anti‐tachycardia pacing (ATP) given in response to VT2 zone [note the last 8/10 beats did not fulfil the morphology match criteria, which induced the ATP therapy]. Note that the same rhythm continues. (C) Another ATP attempt induced ventricular tachycardia (regular tachycardia with EGM wider and different morphology from the previous tracings) with the rate falling in the ventricular fibrillation zone. Note the AT continues independently with AV dissociation. (D) A 25 Joules electrical shock is given, and both atrial and ventricular tachycardia terminate. Ap, atrial paced; As, atrial sensed; F, ventricular fibrillation zone; HV, high voltage; STIM, anti‐tachycardia pacing stimulus; T1, ventricular tachycardia 1 zone; T2, ventricular tachycardia 2 zone; Vp, ventricular paced; Vs, ventricular sensed; VS, ventricular sensed event in below therapy zone (normal).

**FIGURE 3:**
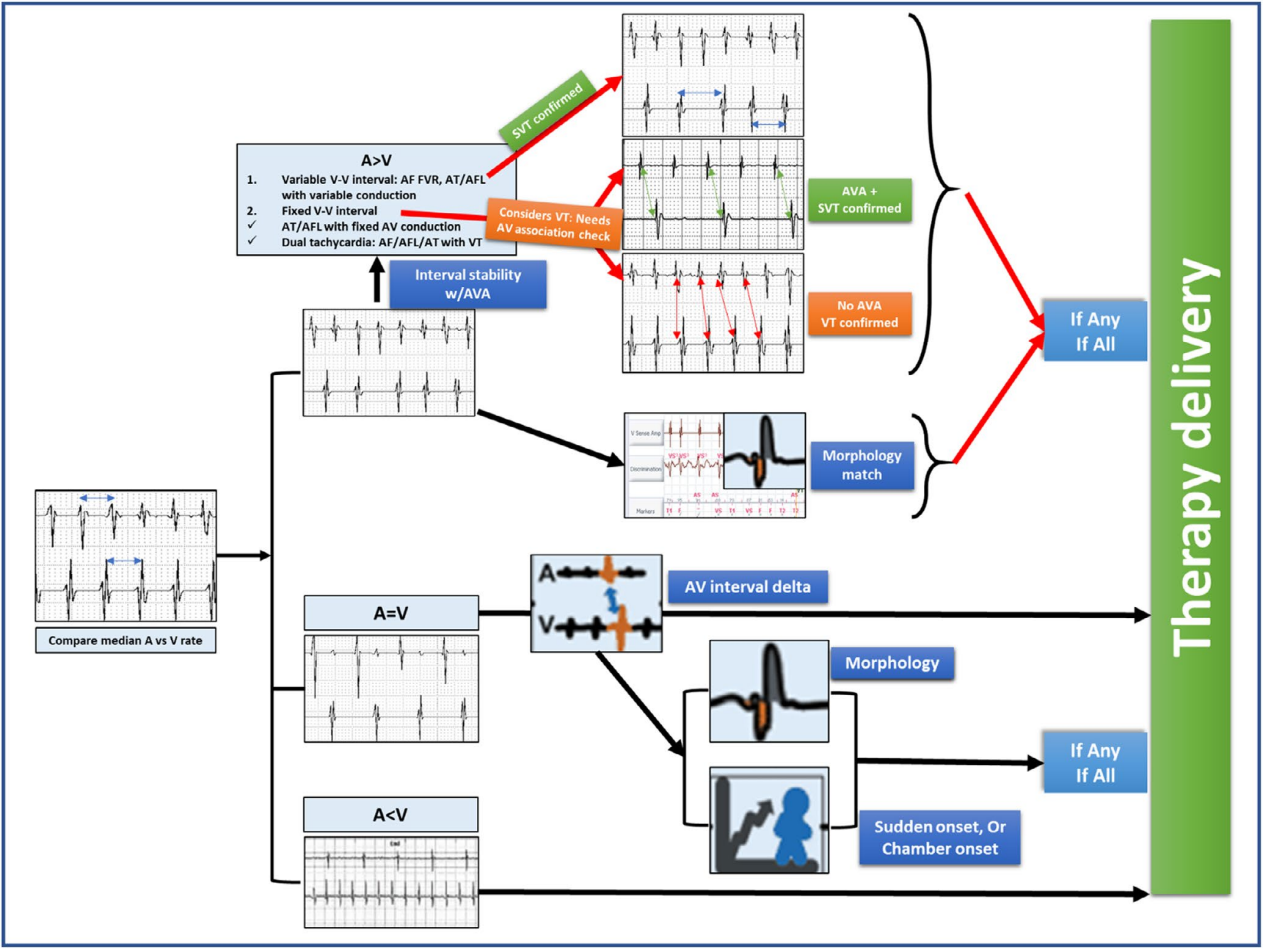
SVT discrimination algorithm in dual‐chamber automated implantable cardioverter defibrillator showing rate branch classification. SVT, supraventricular tachycardia.

Dual tachycardia is defined by the simultaneous or alternating presence of two distinct tachyarrhythmias. Although atrial tachyarrhythmia leading to the induction of ventricular tachyarrhythmias is not uncommon, the induction of VT resulting from inappropriate ATP is rare.^1^ Kalra et al. reported 2 cases of inappropriate ATP therapy for AT and sinus tachycardia out of 79 cases of children with congenital heart disease. Of those two cases, ATP induced VT in AT, which was terminated with shock therapy.^2^ The second case received multiple shocks leading to therapy drain. ATP disrupts reentrant activity, likely by inhibiting conduction within a reentrant circuit. However, in the presence of multiple potential reentry pathways, electrical pacing may modify the existing reentrant circuit path or initiate reentry within a different circuit, thereby altering the characteristics or initiating the VT. Fast VT, a higher number of ATP bursts, scanning, ramp pacing, and a lower left ventricular ejection fraction are associated with a higher likelihood of VT acceleration.^3^, ^4^ Awad et al. reported an occurrence of 8.5% VT acceleration by ATP in their study. They also found that a VT cycle length of less than 310 ms can predict VT acceleration with 76% sensitivity, 68% specificity, 45% positive predictive value, and 86% negative predictive value.^4^ ATP‐induced VTs were associated with a higher risk of VT recurrence.^5^ However, the absence of such events during follow‐up suggests that ATP‐induced VTs may serve as a marker for an underlying arrhythmogenic substrate rather than a direct cause of adverse outcomes.

The index case was planned for atrial flutter ablation. However, the patient preferred to be on aggressive medical management. Once we closely observe the tracings, it can be noted that morphology match violates only when the ventricular rate falls at around 240–300 ms cycle length. This gives the opportunity to escalate the antiarrhythmic therapy by prolonging the AV conduction thereby preventing 1:1 conduction at faster rate as well as reducing the atrial rate [thereby increasing the tachycardia cycle length by reducing automaticity or by reducing velocity in a reentrant tachycardia] in case of recurrence in addition to reducing the recurrence of the atrial arrhythmia itself. Another intervention of changing the settings of “if any” to “if all” was thought of to increase the specificity at the cost of sensitivity. This has the potential hazard of undertreatment of a VT in the presence of dual tachycardia with near similar atrial and ventricular (A > V) rate (which would maintain an apparent AV association maintaining a cut‐off value below 60 ms [AV association delta] and label it as SVT withholding the therapy), the risk of which is high in this clinical setting of ischemic heart disease. The patient has no recurrence of atrial arrhythmia or VT at 6‐month follow‐up on low‐dose amiodarone and optimal beta‐blockers. Another concern of a prolonged atrial arrhythmia being inappropriately treated with shock therapy is the development of thromboembolic episodes unless adequately anticoagulated. Hence, optimum anticoagulation had been ensured for the patient as well.

## AUTHOR CONTRIBUTIONS

Sudipta Mondal: Conceptualization (equal); formal analysis (lead); writing – original draft (lead); writing – review & editing (lead). Swasthi S. Kumar and Jyothi Vijay: Conceptualization (equal); formal analysis (lead); writing – original draft (lead); writing – review & editing (lead). Narayanan Namboodiri: Conceptualization (equal); formal analysis (lead); writing – review & editing (lead).

## CONFLICT OF INTEREST STATEMENT

The authors declare no conflicts of interest.

## PATIENT CONSENT FOR PUBLICATION

Obtained.

## Supporting information


**Figure S1.** Therapy and SVT discrimination programming parameters.

**File S1.** The index event electrogram tracings.

## Data Availability

All data are incorporated into the article and its online supplementary material.
