# Supplementary material for: Once a saint, now a sinner: An appropriate or inappropriate shock?
Source: J Arrhythm. 2025 Jan 10;41(1):e13209. doi: 10.1002/joa3.13209 (PMC11730734; doi:10.1002/joa3.13209)

Episode: VT-2 (210 min-1 / 285 ms)

4 May 2024 10:00 am

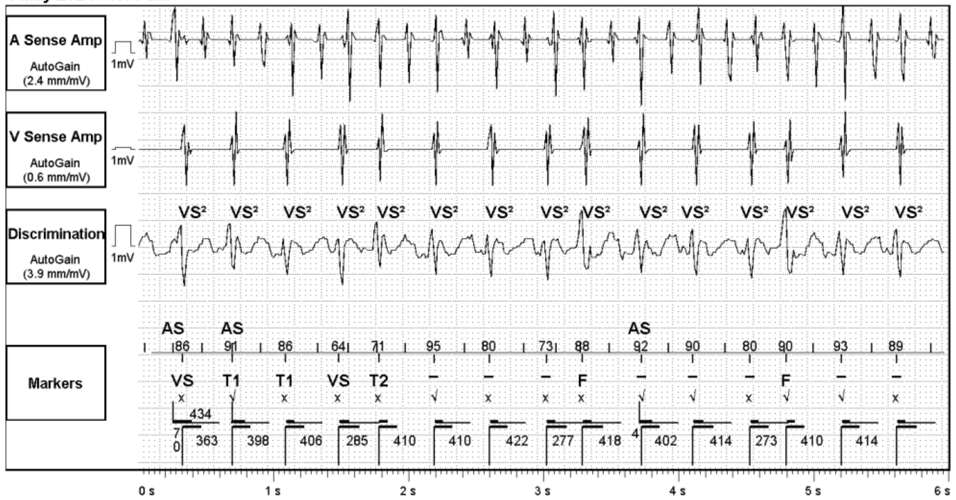

1: A Sense Amp AutoGain (2.4 mm/mV)
2: V Sense Amp AutoGain (0.6 mm/mV)
3: Discrimination AutoGain (3.9 mm/mV)
4: Markers

Sweep Speed: 25 mm/s

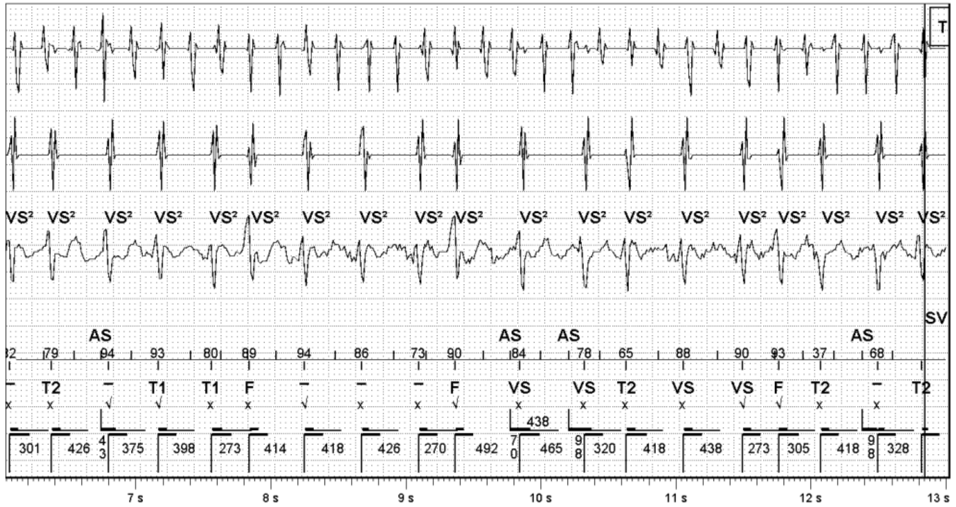

Episode: VT-2 (210 min-1 / 285 ms) (Continued)

4 May 2024 10:00 am

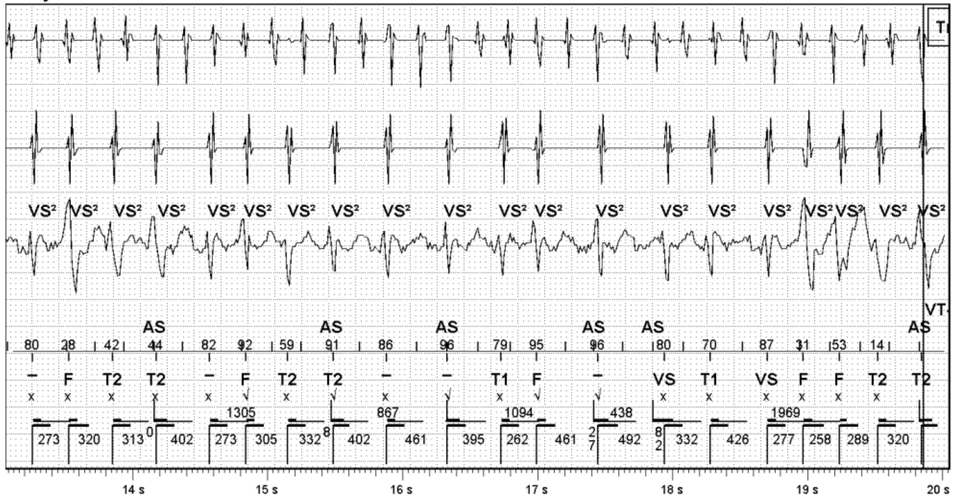

1: A Sense Amp AutoGain (2.4 mm/mV)
2: V Sense Amp AutoGain (0.6 mm/mV)
3: Discrimination AutoGain (3.9 mm/mV)
4: Markers

Sweep Speed: 25 mm/s

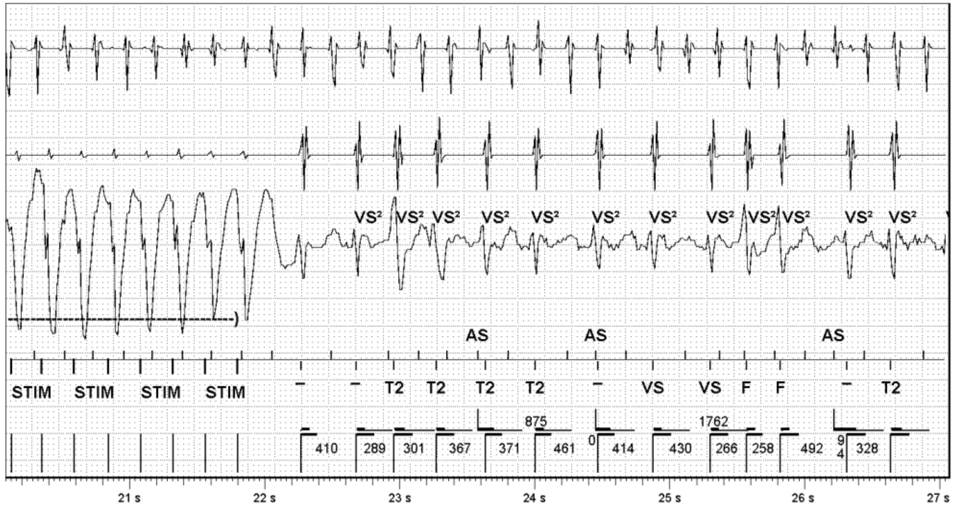

Episode: VT-2 (210 min^{-1} / 285 ms) (Continued)

4 May 2024 10:00 am

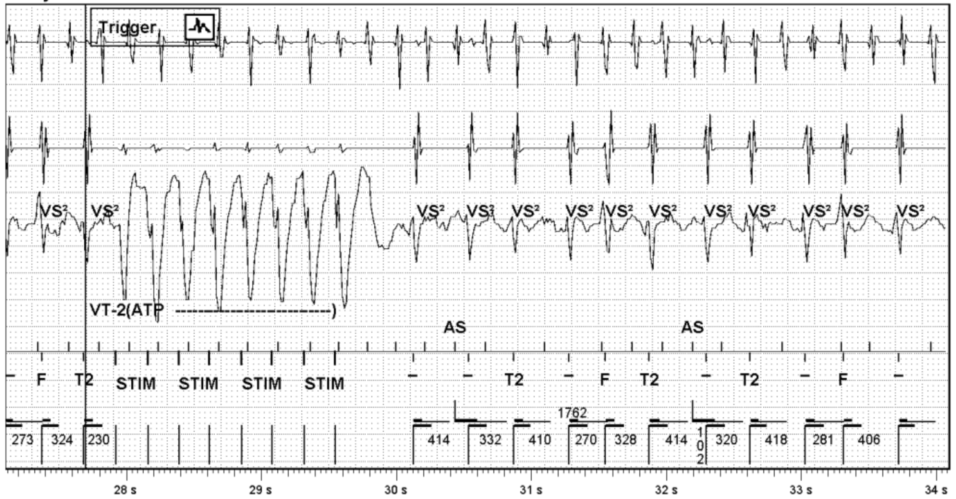

1: A Sense Amp AutoGain (2.4 mm/mV)
2: V Sense Amp AutoGain (0.6 mm/mV)
3: Discrimination AutoGain (3.9 mm/mV)
4: Markers

Sweep Speed: 25 mm/s

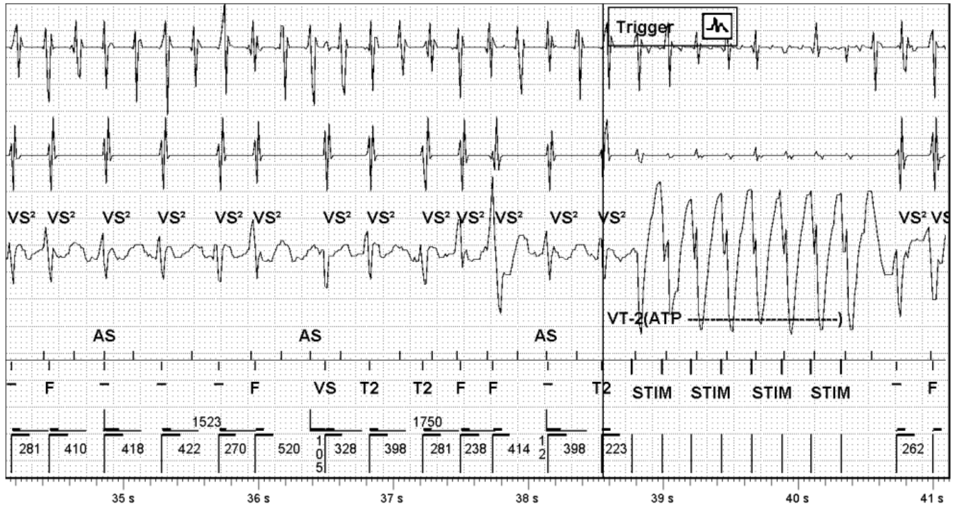

Episode: VT-2 (210 min-1 / 285 ms) (Continued)

4 May 2024 10:00 am

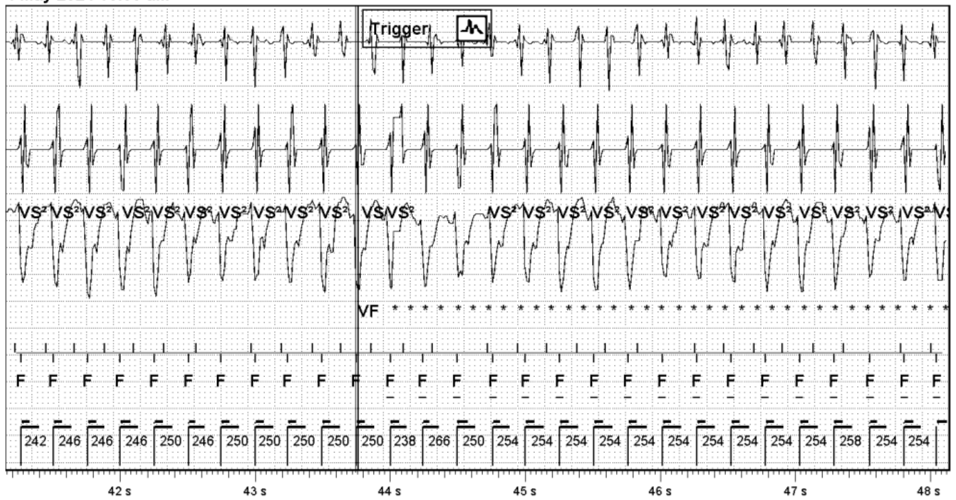

1: A Sense Amp AutoGain (2.7 mm/mV)
2: V Sense Amp AutoGain (0.7 mm/mV)
3: Discrimination AutoGain (2.2 mm/mV)
4: Markers

Sweep Speed: 25 mm/s

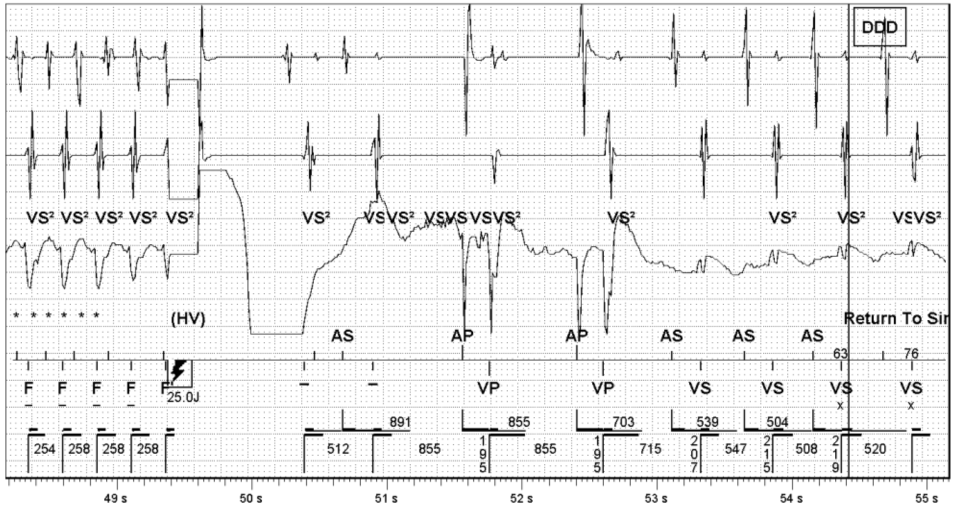

Supplement: Supplementary file 2 — File S1. The index event electrogram tracings. [file JOA3-41-e13209-s001.pdf]
